# Patterns of response and drug involved in patients with multiple drug hypersensitivity syndrome

**DOI:** 10.1186/2045-7022-4-S3-P138

**Published:** 2014-07-18

**Authors:** Inmaculada Doña, Francisca Gomez, Maria Salas, Maria Jose Torres, Carmen Rondon, Paloma Campo, Maria Dolores Ruiz, Leticia Herrero, Maria Auxiliadora Guerrero, Miguel Blanca

**Affiliations:** 1 Regional University Hospital of Malaga, Allergy unit, Spain

## Background

Multiple drug hypersensitivity (MDH) has been defined as a hypersensitivity to two or more chemically unrelated drugs. This has been specially studied in IgE-mediated hypersensitivity reactions to antibiotics and more recently in T-cell-mediated reactions. However, studies focusing in MDH in large populations are lacking. The aim of our study was to describe a well-characterized group of patients diagnosed of MDH.

## Methods

We analyzed retrospectively all patients with a confirmed diagnosis of drug hypersensitivity evaluated in our allergy department between January 2005 and December 2010.

## Results

A MDH was diagnosed in 48 (2.41%) of the 1989 patients evaluated, being 32 females, with a mean age of 50±14,43 years. A total of 137 episodes were reported: 80 (58.39%) suggested an IgE-mediated reaction, 37 (27%) a non-immunologic mechanism (cross-reactive to NSAIDs) and 20 (14.55%) a T-cell mediated reaction. The percentage of MHD in patients with IgE-mediated reactions (9.78%) was higher compared to those with T-cell mediated reactions (5.23%) and non-immunologic reactions (1.91%) (p<0,0001). The drugs most frequently involved were dypirone (13.6%), ciprofloxacin (12.1%), amoxicillin-clavulanic acid (11.4%), amoxicillin (10%), ASA (8.6%), ibuprofen (7.1%) and moxifloxacin (5.7%). Sensitivity to 2 chemically unrelated drugs was diagnosed in 44 patients and to 3 drugs in 4. The most frequent clinical entities were anaphylaxis/shock (42.85%) and urticaria (34.92%).

## Conclusions

Patients with IgE-mediated reactions have a higher risk for developing MHD. More studies are needed to confirm this finding.

